# Nodding Syndrome: A Scoping Review

**DOI:** 10.3390/tropicalmed6040211

**Published:** 2021-12-11

**Authors:** Gasim Omer Elkhalifa Abd-Elfarag, Arthur Wouter Dante Edridge, René Spijker, Mohamed Boy Sebit, Michaël B. van Hensbroek

**Affiliations:** 1 Amsterdam Center for Global Health, Department of Pediatrics and Department of Global Health, Amsterdam UMC, 1105 AZ Amsterdam, The Netherlands; a.w.edridge@amsterdamumc.nl (A.W.D.E.); m.boele@amsterdamumc.nl (M.B.v.H.); 2 Laboratory of Experimental Virology, Department of Medical Microbiology, Amsterdam UMC, 1105 AZ Amsterdam, The Netherlands; 3 Amsterdam Public Health, Medical Library, Amsterdam UMC, 1105 AZ Amsterdam, The Netherlands; r.spijker@amsterdamumc.nl; 4 Department of Psychiatry, College of Medicine, University of Juba, Juba P.O. Box 82, Sudan; mbsebit@gmail.com

**Keywords:** nodding syndrome, disease, sub-Saharan Africa

## Abstract

Nodding syndrome (NS) is a debilitating yet often neglected neurological disease affecting thousands of children in several sub-Saharan African countries. The cause of NS remains unknown, and effective treatment options are lacking. Moreover, knowledge regarding NS is scarce and is based on a limited number of publications, with no comprehensive overview published to date. Therefore, the aim of this scoping review was to summarise the current evidence and identify existing knowledge gaps in order to help clinicians, scientists, and policymakers develop guidelines for prioritising this severe condition. We searched the Medline (Ovid), Embase (Ovid), Scopus, and Global Health Library databases in accordance with the PRISMA extension for scoping review guidance and in accordance with the Joanna Briggs Institute guidelines and methodology for a scoping review, using keywords describing NS. We then extracted and presented the original data regarding the epidemiology, aetiology, pathophysiology, clinical features, diagnosis, management, and outcomes of NS, as well as community perceptions and the psychosocial and economic impact of NS. Out of 1470 identified articles, a total of 69 were included in this scoping review. Major gaps exist in understanding the aetiology and pathogenesis of NS. Future research is urgently needed not only to address these gaps, but also to study the treatment options, epidemiology, and psychosocial and economic impacts of NS. Innovative interventions and rehabilitation programmes designed to address the psychosocial and economic burdens associated with NS are also urgently needed.

## 1. Introduction

Nodding syndrome (NS) is a devastating but often neglected neurological condition that affects thousands of individuals in remote and resource-poor regions in several countries throughout sub-Saharan Africa, with major public health, psychosocial, and economic consequences [1,2,3,4].

Our current knowledge regarding NS is based on data dispersed among scientific articles, viewpoints, health reports, and case reports; however, no comprehensive systematic review of NS has been published to date. Although a few reviews do exist [1,5,6], there is a need for an up-to-date and comprehensive summary of the existing evidence. Thus, the aim of this scoping review was to comprehensively identify, categorise, and summarise the most up-to-date evidence and knowledge gaps regarding NS in order to help clinicians, scientists, and policymakers prioritise this severe yet neglected disease.

## 2. Methods

We performed a scoping review covering the following six domains: (1) epidemiology of NS; (2) aetiology of NS; (3) pathophysiology of NS; (4) clinical features of NS; (5) diagnosis, treatment, and outcomes of NS; and (6) community perceptions and the psychosocial and economic impact of NS. The study was performed in accordance with the PRISMA extension guidelines, as well as guidelines and methods established by the Joanna Briggs Institute for a scoping review as reported by Arksey and O’Malley [7,8] and Levac et al. [7,8].

### 2.1. Inclusion and Exclusion Criteria

Because no clinical definition of NS existed prior to 2012, we included all studies that reported persons with characteristic head-nodding seizures that currently fit the definition of NS, regardless of the individual’s age, country, or year of publication (Table 1). Studies that included epilepsy or onchocerciasis-associated epilepsy (OAE) describing NS patients as a subset were also included if the data regarding the NS subgroup were presented separately. Studies that exclusively described persons with diagnosed epilepsy of non-NS origin and/or other known neurodegenerative diseases were excluded. 

### 2.2. Systematic Identification of Relevant Studies

We systematically searched the Medline (Ovid), Embase (Ovid), Scopus, and Global Health Library databases for articles published from January 1946 to November 2021 (Table 2). We also identified previously unidentified and/or ongoing studies by searching Google Scholar, the study inventories of the World Health Organization, and the US Centres for Disease Control and Prevention (CDC) website. Additional publications were identified using the ‘snowballing’ method (i.e., searching the reference lists in all included studies). 

### 2.3. Selection of Relevant Studies, Data Extraction, and Reporting of Results 

After removing duplicate records, authors GA and AE screened the titles and abstracts of all retrieved articles, excluded any that did not satisfy the inclusion and exclusion criteria, and then performed a full-text screen of the remaining articles. Any disagreements between GA and AE were resolved through discussion with all authors. GA then extracted the data into predefined outcome tables for each of the six outcomes, and AE reviewed 20% of the extracted data (selected at random) for accuracy. Statistical values such as odds ratios and *p*-values were not recalculated. The results were then summarised in tables and figures where possible, and a narrative synthesis was used to describe all other findings.

## 3. Results

**Figure 1:**
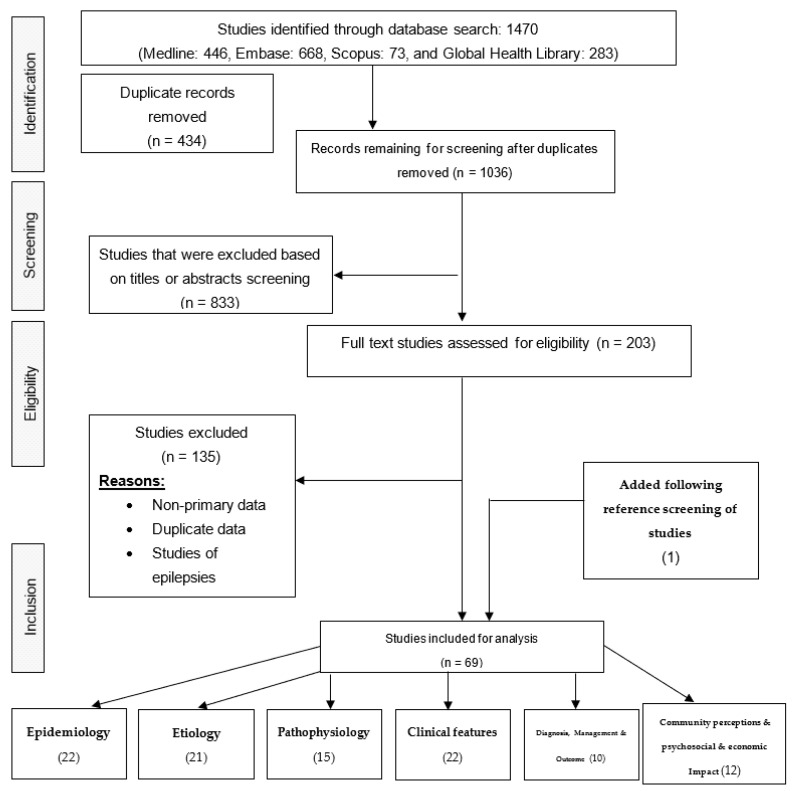
PRISMA flow chart depicting the search strategy and selection of studies. Note that the number of studies that reported all six outcomes totals more than 69, as some studies reported multiple outcomes.

**Figure 2:**
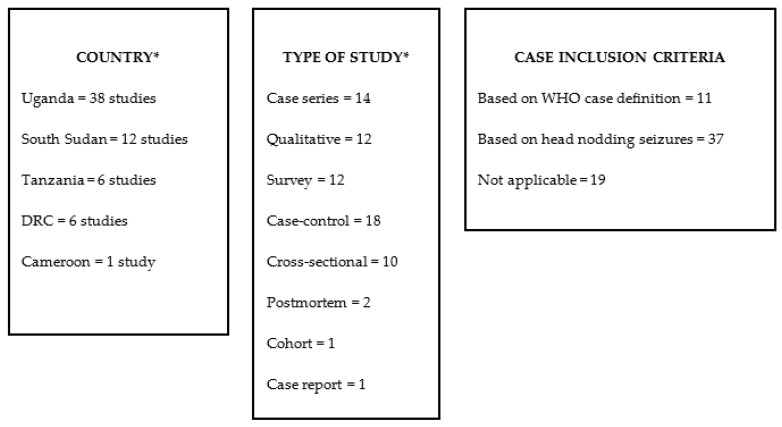
Overview of the study characteristics. * These numbers total more than the number of included papers, as some papers reported data for more than one country or reported more than one study type. DRC: Democratic Republic of Congo; WHO: World Health Organization.

The initial literature search identified a total of 1470 published articles. After duplicate records were removed, 1036 articles remained, 833 of which were excluded after screening the title and/or abstract. After full-text screening of the remaining 203 articles and adding one additional study based on screening the references, a total of 69 articles met the inclusion criteria and were included in our review (Figure 1 and Figure 2). 

### 3.1. Epidemiology 

**Figure 3:**
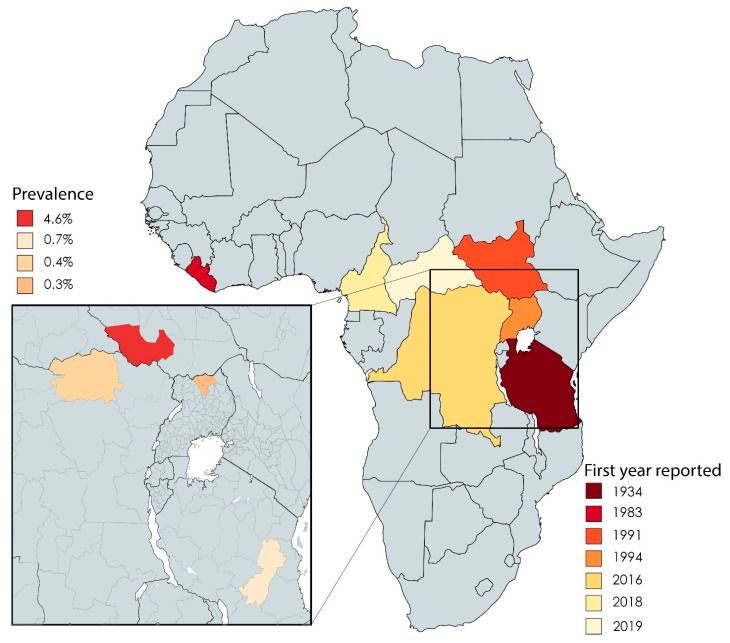
African countries in which cases of NS have been reported, including the first year in which they were reported. The inset shows the reported prevalence of NS in the indicated countries.

Twenty-two studies in our dataset reported on the epidemiology of NS. The first reports of NS cases came from southern Tanzania between the 1930s and the 1960s [10]. These early reports were followed decades later by reports from Liberia [11], southern Sudan (now officially known as the Republic of South Sudan) [3,12], western Uganda [13], and northern Uganda [14] (Figure 3). Recently, NS was reported in regions within the Democratic Republic of Congo (DRC) [15], Cameroon, and the Central African Republic [16,17] (Figure 3). 

On a regional level, the overall prevalence of NS was reported to be 4.6%, 0.7%, 0.4%, and 0.3% for South Sudan (Western Equatoria) [3,18], northern Uganda (Kitgum, Pader, and Lamwu districts) [19], the DRC (town of Aketi) [20], and Tanzania (Ulang district and Morogoro region) [21], respectively. In Cameroon (Bilomo, Kelleng, Ngongol, Nyamongo, and Bayomen), the reported prevalence of NS among a subgroup of patients with epilepsy was 21.8% [16]. Finally, in the Central African Republic, a total of five cases were identified among 6175 individuals [17].

In villages, NS has been reported to cluster around rapidly flowing rivers infested with blackflies (*Simulium* spp.) [12,15,16,18,20,22,23,24,25] and in families, with some families having two or more affected members [3,10,19,26,27,28,29]. In addition, NS has also been associated with poverty, food shortage, and a history of displacement [12,18,23,25].

### 3.2. Aetiology 

The aetiology of NS was investigated and reported by 18 studies in our dataset, which were further subdivided into the following seven categories (with some studies reporting more than one category): infections (*n* = 11), nutritional deficiencies (*n* = 4), toxins (*n* = 5), and autoimmune (*n* = 4), hormonal (*n* = 3), metabolic (*n* = 2), and genetic factors (*n* = 1). These categories are discussed in detail below.

#### 3.2.1. Infection 

With respect to parasitic infection, most studies focused on *Onchocerca volvulus* (OV) infection (Table 3) and studied the association between NS and OV infection using skin snip microscopy, serology (Ov16 IgG detection using ELISA and OvFAR/MSA detection using the luciferase immunoprecipitation system), and PCR analysis. We found that four case–control studies using skin snip microscopy measured a significantly higher prevalence of OV infection in NS cases (range: 71.1–96.7%) compared to controls (range: 43.7–53.9%) [12,18,30,31]. In addition, one case study using skin snip microscopy found a higher density of OV microfilaria in NS cases compared to patients with other forms of epilepsy [32]. In contrast, case studies using PCR on cerebrospinal fluid (CSF) samples did not detect either genomic material of OV in 139 cases [12,29,33,34] or species of *Wolbachia* (an endosymbiotic bacteria that occurs together with OV) in CSF samples taken from 10 NS cases [33]. In addition, *Mansonella perstans* infection, which was detected using microscopy on blood samples, was significantly associated with NS in one case–control study, with an odds ratio (OR) of 3.2 (*p* = 0.005) [12] (Table 3). Finally, we found no association between NS and the presence of any other parasitic infections, including *Loa loa*, *Wuchereria bancrofti*, *Trypanosoma gambiense* (a protozoan that causes human African trypanosomiasis, or sleeping sickness), and *Taenia solium* (an intestinal tapeworm that causes cysticercosis) [12,31]. 

With respect to viruses, three case–control studies investigated measles infection using a previous history of measles infection as reported by the parents or legal guardians, with conflicting results (Table 3). The first study (conducted in South Sudan in 2002) reported an inverse association between prior measles infection and NS (OR: 0.13; *p* = 0.025) [12]. The second study (conducted in Uganda in 2009) found no significant association (OR: 3.3; 95% CI: 0.8–13.6) [31], and the third study (also conducted in Uganda, but in 2014) found a significant positive association between measles infection and NS (OR: 6; *p* = 0.047) [35]. In addition, no association was identified between *hepatitis E* infection and NS [31]. Finally, a recent case–control study found no association between NS and known viruses including *Anneloviridae*, *Hepadnaviridae (hepatitis B)*, *Flaviviridae*, *Herpesviridae*, *Polyomaviridae* (human polyomavirus), *Papillomaviridae*, and *Virgaviridae* [36]. 

Our search revealed no studies regarding bacterial infections. Finally, there was no association between prion disease and NS (based on a reported history of eating monkey meat) [31].

#### 3.2.2. Nutritional Deficiency 

A case–control study in Uganda found that compared to controls, NS cases had lower plasma levels of vitamin B6 (OR: 7.2; *p* = 0.001) and higher plasma 3-hydroxykynurenine levels (OR: 4.50; *p* = 0.013) [38] (Table 4). In contrast, however, three other studies conducted in Uganda and Tanzania found no such associations [31,39,40]. 

No significant association was found between NS and other micronutrient deficiencies, including vitamin A, vitamin B12, folate, zinc, and selenium [31]. 

#### 3.2.3. Toxins

With respect to food-related toxins, a history of consuming maize (OR: 4.0; *p* = 0.05), mouldy maize (OR: 4.3; *p* = 0.009) [35], relief foods (OR: 4.0; *p* = 0.02) [35,38], and either red or brown sorghum (OR: 6.22; *p* = 0.05) [12] was significantly associated with NS (Table 5). One study also investigated the possible underlying aetiology associated with consuming mouldy foods by examining the presence of various mycotoxins (aflatoxin, ochratoxin, and ribotoxin deoxynivalenol) present in contaminated foods (maize, sorghum, millet, and groundnuts), but found no association [41]. 

Another case–control study failed to confirm the association between red/brown sorghum and NS [31]. Interestingly, one study reported an association—albeit not significant—between NS and a history of eating baboon meat [18], whereas other studies found no association between NS and the consumption of agricultural seeds, cassava, river fish, insects, rodent meat, or bush meat (including brains) [12,31]. Finally, one case–control study detected the mycotoxins α-zearalenone, aflatoxin M1, and T-2 toxin in the urine of both cases and controls, but found no significant difference between cases and controls [42].

Aside from food-related toxins, one study in Uganda found a positive association between NS and the use of crushed plant roots as traditional medicines (OR: 5.4; 95% CI: 1.3–22.1) [31] (Table 5). However, another study conducted in the same area found no such association (OR: 1.29; 95% CI: 0.47–3.6) [35]. Exposure to toxins from war munitions was associated with NS in Uganda (OR: 13.9; 95% CI: 1.4–135) [31], but not in South Sudan [12,30]. Finally, no association was found between NS and exposure to copper or mercury and the source of water for domestic use (river, borehole, spring, shallow well, or pipe) [31].

#### 3.2.4. Autoimmunity 

One case–control study used a protein array to screen for a large number of autoantibodies, revealing that autoantibodies against the protein leiomodin-1 had the strongest association with NS; specifically, leiomodin-1 autoantibodies were found in 53% of NS cases compared to 31% of controls (OR: 2.7; 95% CI: 1.1–6.5) [43]. However, a recent study could not confirm the association between autoantibodies against leiomodin-1 and NS [44]. 

One study found no autoantibodies against *N*-methyl-D-aspartic acid (NMDA) receptors or the voltage-gated potassium channel (VGKC) complex in the serum of any NS cases tested [40].

Finally, another case–control study found that the mean plasma levels of macrophage migration inhibitory factor (MIF) were significantly elevated (47.3 ± 25 ng/mL vs. 17.8 ± 6 ng/mL) in NS cases compared to the healthy controls, which was hypothesized to play a role in the development and disease progression as a result of autoimmunity and neuroinflammation [45]. However, the frequency of MIF −173 C genotypes (CC/CG) was significantly lower in NS cases compared to the healthy controls (OR 0.33; 95% CI 0.14–0.8) [45]. 

#### 3.2.5. Hormonal, Metabolic, and Genetic Factors

With respect to hormonal imbalances, a case series of Ugandan adolescents reported low levels of the peptide hormone somatomedin C (also known as insulin-like growth factor-1) in two out of eight NS cases. This study also reported low levels of luteinising hormone, follicle-stimulating hormone, and the sex hormones testosterone and oestrogen in seven of eight cases [46]. In contrast, all eight cases had normal levels of other hormones, including thyroid hormone, parathyroid hormone, growth hormone, adrenocorticotropic hormone, adrenal corticosteroids, and mineralocorticoids [46,47]. Finally, a case–control study found no association between serum serotonin level and NS [48]. 

Altered metabolism was identified as a possible cause of NS by a case series involving 48 Ugandan patients with low mean levels of biotinidase and acetyl carnitine, but normal urate levels [49]. In addition, another case series involving 10 Ugandans reported high anion gap metabolic acidosis [47]. 

With respect to genetic aetiological factors, one case–control study in South Sudan (with 48 cases and 51 controls) found that the presence of specific amino acids at specific positions in the HLA-B (Ala11, Ala24, Asn63, and Phe67), DRB1 (Ala73 and Thr77), and DQB1 (Pro56, Glu66, and Val67) proteins were significantly associated with increased susceptibility to NS, while other specific amino acids in the HLA-B (Ser11 and Glu63), HLA-C (Trp156 and Glu163), DRB1 (Lys71, Gly73, Arg74, and Asn77), and DQB1 (Leu56, Asp66, Ile67, Glu70, and Asp71) proteins were associated with a decreased risk of NS [50]. A separate, relatively small case–control study (with three cases and five controls) of Ugandan children reported normal mitochondrial DNA and negative oligonucleotide microassay results for consanguinity, deletion, and duplication [39]. 

### 3.3. Pathophysiology 

One autopsy study of five deceased NS cases in Uganda suggested that NS may represent a new form of tauopathy based on the presence of tau-immunoreactive neuronal neurofibrillary tangles, pre-tangles, neuropil threads, and dot-like tau in the cerebral cortex, brain stem, and basal ganglia of all five cases [51]. However, another autopsy study of another five Ugandan cases could not confirm these findings; instead, the authors proposed a neuroinflammatory pathophysiology, as they found no signs of generalised tauopathy, but rather found cerebellar atrophy, a loss of cerebellar Purkinje cells, cortical gliosis, and features indicating previous ventriculitis and/or meningitis [52]. This conclusion based on neuroinflammation was consistent with the findings of six separate magnetic resonance imaging (MRI) studies involving 66 cases, which found gliosis (mainly in the occipital and parieto-occipital areas) and cerebellar atrophy, with no focal changes in the cerebral cortex or hippocampus [4,14,29,34,39,53]. This neuroinflammation did not appear to be the result of a chronic infection in the central nervous system (CNS), as six studies (with a total of 148 cases) reported normal cell counts, protein levels, and glucose concentration in the CSF [4,14,29,31,33,39]. Interestingly, one of these six studies reported the presence of oligoclonal bands in the CSF—which indicates the presence of immunoglobulins that are commonly found in a wide range of neuroinflammatory diseases—in one of the three cases in their study [39]. 

No cytokine-specific profile was identified in any of the NS cases; however, one case–control study reported higher levels of the cytokine-like polypeptide C5a in the CSF of NS cases compared to controls, suggesting complement activation [54]. The same study also found lower plasma levels of CCL2, CCL5, CXCL13, IL-10, APRIL (also known as TNFSF13), and MMP-9 (matrix metallopeptidase 9) in NS cases compared to controls [54]. Another study reported decreased levels of IL-13 in NS cases, but could not detect IL-5, IL-6, or TNF-α [55]. 

Seven studies (with a total of 111 cases) reported findings based on electroencephalogram (EEG) recordings and found evidence of atonic seizures (i.e., presence of electro-decrement and paraspinal electromyographic dropout) in patients who experienced nodding episodes during the EEG recordings [14,53]. In addition, EEG recordings also showed evidence of generalised and focal seizures [4,14,18,26,29,39,53].

With respect to nonspecific laboratory findings, several case series reported low haemoglobin levels [4,10,47], elevated eosinophil counts [4,29], and increased erythrocyte sedimentation rates [4] in NS cases. In contrast, liver function, kidney function, white blood cell counts, and platelet counts were all normal [4,29,47]. 

### 3.4. Clinical Features 

Prior to the onset of typical head-nodding seizures, 20% of NS cases seem to develop prodromal signs, including an expressionless stare, excessive sleepiness, dizziness, and loss of attention [4,30,56]. The characteristic involuntary repetitive head nodding—which may be present in all NS cases—likely results from a periodic loss of neck muscle tone [13,14,18,30]. The onset of these symptoms has been described in both children and young adults ranging from 2 to 22 years of age, with a slightly higher prevalence among males (55%) [4,10,13,14,16,19,25,26,31]. In the majority of cases, head nodding is reported to be triggered by the sight of food or by cold weather [4,14,16,18,29,30,46,57]. 

In over 80% of cases, NS progresses to include other seizure types, including generalised tonic–clonic seizures, partial complex seizures, and atypical absence seizures [4,12,14,16,18,21,26,29,53,56]. In addition, approximately 30% of patients develop cognitive impairment, which can present as a slow reaction time, depression, and/or dropping out of school [4,12,13,14,16,18,24,26,30,56,57,58]. Impaired physical development is also reported, including stunted growth with delayed bone age (41% of cases), wasting (73% of cases), and delayed puberty [4,13,16,24,46,56,58,59]. 

Further complications reported to be associated with NS include psychiatric symptoms such as mood changes, aggressiveness, sleep disturbances, and wandering in 36%, 27%, 23%, and 9% of cases, respectively, as well as catatonia symptoms (e.g., staring, mutism, stupor, and grimacing) in some cases [4,58,60]. In contrast, neither focal neurological abnormalities nor cranial nerve palsies have been reported in NS [4,13,14,30].

Some patients were described to progress to even more severe forms of NS, although the percentage of patients who do so is currently unknown. Children with a severe form of NS are often severely mentally retarded with impaired speech or a complete loss of speech, the inability to stand, urinary incontinence, a tendency to wander, and Parkinson-like features such as drooling, facial masking, facial tics when speaking, and slow speech patterns [4,10]. These patients may die due to uncontrolled generalised seizures or other events such as falling into the fire while cooking or drowning [4,10,12,13,14,18,29,56,58]. To date, no reports of children making a full recovery from NS have been published.

### 3.5. Diagnosis and Management

Currently, no laboratory test is available to confirm the clinical diagnosis of NS. However, in 2012 a consensus case definition was established during the International Scientific Meeting on Nodding Syndrome held in Kampala, Uganda [9], which was modified in 2013 during the single-stage cluster survey conducted by the CDC and the Ugandan Ministry of Health to determine the prevalence of NS in Uganda (Table 6) [19]. This definition is now used widely by clinicians and scientists. Prior to 2012, however, characteristic repetitive head-nodding seizures were used to diagnose NS.

Seven studies in our dataset reported on the management of NS. Five of these studies found that the use of anticonvulsants—including phenobarbitone, carbamazepine, sodium valproate, and phenytoin (either individually or in combination)—reduced seizure frequency in 70% of cases, with complete seizure control achieved in 25% of cases [13,29,53,59,61]. In addition, anticonvulsants were reported to increase basic self-care, behaviour, and school attendance in 80%, 59%, and 40% of cases, respectively, thus reflecting an increase in independence [61]. In one study, the combined use of anticonvulsants and multivitamins was reported to reduce wasting and stunting [59]; however, whether these interventions affect disease progression in NS remains unknown. Finally, community-directed treatment with ivermectin combined with larviciding of rivers was reported to coincide with a reduction in the incidence of NS in northern Uganda [62]. 

### 3.6. Community Perceptions and Psychosocial and Economic Impact 

Twelve qualitative studies (all of which were performed in Uganda) investigated community perceptions and the psychosocial and economic burden of NS. 

#### 3.6.1. Perceptions and Beliefs

Within communities, NS was perceived to be associated with a wide variety of factors, including living in a camp for internally displaced people [63,64,65]; consumption of expired and/or contaminated relief foods [64,65,66]; exposure to chemicals from war munitions [63,64,65,67,68]; evil spirits, being cursed, or punishment from the gods as a result of bad deeds committed during a time of war [58,63,64,65,66,67,69]; open-gold mining in Karamoja region (northeastern Uganda) polluting the rivers with heavy metals such as mercury [65]; and blackflies breeding in the rivers in northern Uganda [63,67]. 

#### 3.6.2. Transmission, Presentation, and Treatment 

In some studies, communities reported the belief that NS can be transmitted from person to person through saliva and the air [58,63,64,66,68]; therefore, patients with NS are often isolated from their family members and peers (e.g., eating and sleeping separately) in NS-affected communities. Some communities reportedly perceive NS as a distinct disease entity from epilepsy, given that NS is generally more severe and can include severe mental and/or physical disabilities [66,68]. Furthermore, local communities in northern Uganda reported believing that NS cannot be cured [58,63,69] and that anticonvulsive medications—particularly sodium valproate—are associated with promiscuous behaviour, resulting in low treatment compliance [70]. 

#### 3.6.3. Health-Seeking Behaviour of Caretakers

Caretakers generally seek healthcare from government health facilities; however, a shortage of healthcare workers, long waiting hours, frequent lack of medicines, and transport-related issues (e.g., a lack of money for transportation and/or difficulties associated with transporting mentally and physically disabled patients with NS) often drives caretakers to seek care from traditional healers and witch doctors [70,71]. 

#### 3.6.4. Psychosocial and Economic Burden

The mental and physical impairments caused by NS often lead to personal and family shame [70,72], discrimination, social isolation [65,66,67,72], and economic constraints due to reduced livelihood activities [66,67,68,72]. In addition, caretakers experience poor sleep and are often depressed, which can lead to domestic violence, substance abuse, and suicidal and homicidal thoughts, potentially ending in family separation [66,67,68,72]. Some caretakers report feeling that their children with NS are useless [65,69]. In addition, children often drop out of school due to fears related to the person-to-person transmission of NS [66,67]. Finally, some individuals with NS report being sexually abused, exploited, or forced to engage in child labour [70].

## 4. Discussion

Nodding syndrome is an unexplained, currently untreatable, and devastating neurological disease currently affecting thousands of individuals in various sub-Saharan African countries. Despite the high burden placed on affected individuals and their families, NS has received strikingly little attention; moreover, the available information is scattered throughout the literature, thus prompting the need for this scoping review in which we summarise the evidence to date and reveal research and knowledge gaps. The paucity of knowledge regarding NS is due primarily to a lack of funding to conduct studies in NS-affected areas, which are often challenging due to access constraints, civil conflicts, poverty, and a lack of trained personnel. In addition, NS has been a largely neglected disease even though it leads to significant physical, mental, and socioeconomic impairments. 

The first cases of NS were reported in Tanzania in the 1930s; today, however, South Sudan is considered to have the highest number of cases [3,10,16,18,19,20,21]. Thus, the epidemiological picture is still changing, with new cases emerging in South Sudan (in Mundri West County and Mundri East County; our unpublished observations), and hardly any new cases reported in southern Tanzania (Mahenge, Ulanga district) [73] and northern Uganda (Kitgum and Pader districts) [62]. However, due to the paucity of large-scale studies, solid epidemiological data from affected countries and regions are not available to support these anecdotal observations or establish the true burden of NS. Interestingly, new cases of NS were reported recently in the DRC (in 2016), Cameroon (in 2018), and the Central African Republic (in 2019) [16,17,20], suggesting that NS may be prevalent in many more countries than previously known.

With respect to the underlying cause of NS, many factors have been associated with NS and suggested as potential causes; however, to date none of these have been shown definitively to cause NS. Nevertheless, the putative association between NS and infection with the filarial nematode *Onchocerca volvulus* (OV) has gained considerable interest. This is not surprising, as this is the only association consistently identified by multiple studies [12,18,30,31]. However, one should be careful not to overinterpret this association or consider it definitive evidence of a causal relationship. From a pathophysiological perspective, it seems unlikely that OV invades the CNS. Although early studies detected small numbers of OV nematodes in the CSF of patients following treatment with the anthelmintic drug diethylcarbamazine [74], the normal biochemical profile of CSF and the inability of recent studies to detect OV or *Wolbachia* filarial endosymbionts in the CSF using microscopy or PCR seems to rule out the likelihood of CNS infection [12,29,33,34]. An alternative pathophysiological mechanism was suggested to involve an autoimmune response induced by OV infection, for example the so-called leomodin-1 hypothesis [43]. Because leiomodin-1 shares high homology with an OV-like protein, and because autoantibodies have been shown in vitro to induce neurotoxicity, a possible mechanism involving molecular mimicry induced by OV infection has been hypothesised. Several findings, however, makes this an unlikely possibility. First, healthy controls living in the same villages as NS cases also have antibodies, at only a marginally lower prevalence. Second, no association has been found between OV status and anti-leiomodin-1 antibody levels. Finally, whether the leomodin-1 protein is localised to the extracellular side of CNS cells has been debated [75,76]. 

On the other hand, the repeated findings of an apparent association between OV infection and NS should not be ignored. For example, given that NS is likely multifactorial, OV infection may be only one piece of the puzzle and may require additional factors such as immunosuppression due to malnutrition, toxins (e.g., mycotoxins), and/or genetic predisposition such as a specific HLA haplotype, as recently suggested [50]. Alternatively, rather than playing a causal role, OV infection may be a confounding factor associated with a yet-to-be-determined cause. For example, NS may be caused by an unidentified neurotropic virus transmitted by the same vector as OV (i.e., blackflies). Another possibility is a converse causal relationship between OV and NS, in which patients with NS may have an increased risk of becoming infected with OV; this is certainly plausible, given that individuals with NS are often isolated, drop out of school, and have a tendency to wander off, thus increasing their exposure to blackflies. Clearly, future studies are needed in order to investigate these causal relationships, as well as potential confounders and the interaction with OV. 

The possible association between prior measles exposure and NS is particularly interesting given certain similarities between NS and subacute sclerosing panencephalitis (SSPE), a chronic progressive brain inflammation occurring years after infection with a hypermutated strain of the measles virus. A previous study suggested an association between NS and SSPE based on the finding that both NS and SSPE affect children at around the same age and have the same gender bias, as well as the finding that NS cases often increase following a peak in measles cases [35]. On the other hand, NS and SSPE differ in several aspects. First, the head nodding in NS is triggered by food and cold weather, whereas the initial myoclonic head jerks in SSPE are triggered by excitement [77]. Second, unlike SSPE, NS does not present with focal neurological or visual impairment [77]. Third, the CSF in children with NS is often normal, while gamma globulin levels are often increased in the CSF in SSPE (representing more than 20% of total CSF proteins) [77]. Fourth, the above-mentioned studies used the reported history of measles infection, which can be prone to many types of bias. In summary, it seems unlikely that NS is caused by SSPE, although further study is clearly needed. In addition, NS may actually be caused by an SSPE-like pathophysiological mechanism caused by a different virus. Given that viral infections have not yet been investigated in detail, this notion warrants further study. 

The reported putative association between low levels of vitamin B6 (specifically, pyridoxal-5-phosphate) and NS [38] is potentially relevant, as severe vitamin B6 deficiency can cause convulsions [78,79]. However, three studies were unable to confirm this association [31,39,40], although one study found that vitamin B6 deficiency was common throughout the entire study population. Low levels of vitamin B6 in NS cases could be a complication of the disease, rather than an aetiological factor. Because humans cannot synthesise vitamin B6, it must be provided by dietary sources; however, patients with NS often have poor eating habits due to sleepiness and because eating can trigger nodding seizures. On the other hand, given that NS does not appear to be associated with deficiencies in vitamin A, vitamin B12, folate, zinc, or selenium, dietary factors are less likely. Future studies are therefore warranted in order to re-evaluate the association between NS and vitamin B6; ideally, such studies should include cases in the early stages of disease in order to minimise the decrease in dietary intake that results from NS. Moreover, vitamin B6 is a complex consisting of six separate vitamers, of which only one (pyridoxal-5-phosphate) has been studied; thus, measuring the levels of all six vitamers in the plasma and CSF may provide new pathophysiological insights. 

Several toxins have also been suggested to play a role in NS; however, the epidemiological data do not appear to support this association. Although Spencer et al. previously suggested that mycotoxins in grains consumed during times of displacement in South Sudan may be associated with NS [12], similar grains were also consumed by other displaced persons in other regions within South Sudan, with no reported cases of NS. Moreover, the same group recently reported that both children with NS and children without NS harbour foodborne mycotoxins [42]. Similarly, the use of crushed plant roots in traditional medicines seems unlikely to be the cause of NS, as they are used widely around the world [80]. Lastly, chemicals from war munitions, which were identified as a possible cause of NS in Uganda, were not found to be associated with NS in South Sudan, which experienced more than 21 years of civil war and never reported a single case of NS in their most war-affected areas, including the Eastern Equatoria State. 

Interestingly, the presence of low average levels of biotinidase and acetyl carnitine detected in a few NS cases in Uganda suggests a possible metabolic cause [49]. However, carnitine deficiency-related encephalopathies are often accompanied by skeletal muscle myopathy and cardiomyopathy, neither of which is typically seen in NS [81]. Similarly, biotinidase deficiency is an unlikely cause of NS, as it usually presents with vision and hearing loss, cutaneous abnormalities, and hypotonia, none of which are typically seen in NS [82]. Nonetheless, a thorough evaluation of metabolic changes is warranted in order to definitively include or exclude their role in the aetiology of NS.

Autopsy studies will likely play an essential role in determining the underlying pathophysiology of NS through examination of postmortem histopathological patterns and confirmation of the antemortem microbiological diagnosis; however, only two autopsy studies have been reported to date. The first study suggested that NS may represent a new form of tauopathy [51], as the result of either the NS disease itself or the underlying repetitive seizure activity [83,84]. In contrast, the second autopsy study concluded that NS may be the result of a generalised neuroinflammatory pathophysiological process [52]. A general limitation of both autopsy studies is the relatively small number of cases (with only five cases each), making it difficult to draw any clear conclusions. Finally, other pathophysiological studies using MRI, EEG, and generalised laboratory tests also failed to provide any insights into the pathophysiological mechanisms underlying NS. 

NS was recently suggested to be part of a spectrum of diseases known as onchocerciasis-associated epilepsy (OAE), which is defined as two or more seizures with no obvious cause occurring between the ages of 3–18 years in a previously healthy person who had lived for at least 3 years in an onchocerciasis-endemic area [57]. NS may indeed be a subtype of OAE, given the similar epidemiology, the fact that both are epileptic disorders, and the fact that both are associated with OV infection. The proposed differential diagnosis of OAE includes NS, Nakalanga syndrome (defined as epilepsy with impaired growth, physical deformities, endocrine dysfunction, and mental impairment), and other forms of epilepsy such as epilepsy in onchocerciasis-endemic areas. However, given that NS has not been shown definitively to fit within the OAE spectrum with a similar pathophysiological mechanism, for the purposes of this scoping review, we limited the evidence to patients with NS.

No cure currently exists for NS. However, based on the relatively few intervention studies conducted to date (albeit without a control group for comparison), the ideal management of NS would likely include prophylactic anticonvulsants, nutritional supplementation, physiotherapy and rehabilitation, and psychosocial support for the patient and his/her family. Such an approach would require a multidisciplinary team of specialists, which is currently difficult to implement in the resource-poor areas in which NS is endemic. On the other hand, community-guided administration of ivermectin combined with application of larvicides to rivers in northern Uganda has been shown to coincide with a reduction in the incidence of NS [62]. However, whether this reduction in NS cases was due to larviciding the rivers (thus reducing blackfly populations), ivermectin treatment (thus reducing OV microfilaria load), and/or another unrelated factor remains an open question. Clearly, carefully controlled studies are warranted in order to explore the possible effect of each of these factors. 

The major limitation in our inclusion criteria was that we only included publications in English language, which excluded any relevant publications in other languages. 

## 5. Conclusions

In light of the relatively limited data reported on NS to date, more studies are needed in order to increase our knowledge regarding this devastating yet often neglected disease. In this respect, large population-based studies spanning sub-Saharan Africa will likely provide a more complete picture of the true burden of NS. In terms of aetiology, future studies should focus on examining a wider range of possible factors, including metabolic, nutritional, autoimmune, genetic, viral, and other infectious agents, as well as potential interactions between these factors. Follow-up studies are also needed in order to differentiate between potential causal associations with OV, confounding factors, and/or other previously associated factors. In addition, studies involving animal models may help to test emerging hypotheses regarding causal factors. Moreover, postmortem studies including larger numbers of well-preserved samples will likely provide valuable information regarding the underlying pathophysiology, and randomised controlled trials are needed in order to evaluate new treatment options such as anticonvulsants and micronutrient supplementation. Finally, innovative programmes designed to address the psychosocial and economic impacts of NS, as well as rehabilitation strategies, are urgently needed. 

## Figures and Tables

**Table 1:** Inclusion and exclusion criteria.

	Inclusion Criteria	Exclusion Criteria
Study design and population	All study designs of persons with nodding syndrome (according to clinical case definition [9] or cases with features of nodding syndrome) of any age	Studies of known epilepsies other than NS and other known neurodegenerative diseases
Publications	All publications: Commentaries, editorials, letters, books, book chapters, dissertations, and conference proceedings with primary/original study data	Studies with nonprimary data
Outcome	Epidemiology, aetiology, pathophysiology, clinical features, diagnosis, and treatment of NS; community perceptions and the socioeconomic impact of NS	
Language, year, and country	English publications from 1946 onward, from any country	

**Table 2:** Search strategy.

Database	Search Terms
Embase	#1. ‘nodding syndrome’/exp #2. (nodding NEAR/2 (head OR disease OR syndrome)):ti,ab,kw #3. river epilepsy’:ti,ab,kw #4. onchocerciasis’/exp OR ‘onchocerca’/exp OR onchocerc *:ti,ab,kw #5. epilepsy’/exp OR nodding:ti,ab,kw OR epilepsy *:ti,ab,kw OR seizure *:ti,ab,kw #6. #4 AND #5 #7. #1 OR #2 OR #3 OR #6
Medline	#1. exp Nodding Syndrome/ #2. (nodding adj2 (head or disease or syndrome)).ti,ab,kf. #3. river epilepsy.ti,ab,kf. #4. exp Onchocerciasis/or exp Onchocerca/or onchocerc *.ti,ab,kf. #5. exp Epilepsy/or (nodding or epilepsy * or seizure *).ti,ab,kf. #6. 4 and 5 #7. 1 or 2 or 3 or 6
Scopus	TITLE-ABS-KEY ((nodding AND near/2 (head OR disease OR syndrome)) OR ‘river AND epilepsy’ OR (onchocerc* AND (nodding OR epileps* OR seizure*)))
Global Health Library	ab:(nodding AND (disease OR syndrome OR head) OR ‘river epilepsy’ OR (onchocerc * and (nodding or epilepsy * or seizure *))) ti:((nodding AND (disease OR syndrome OR head) OR ‘river epilepsy’ OR (onchocerc * and (nodding or epilepsy * or seizure *))))

**Table 3:** Case–control studies reporting associations between pathogens and nodding syndrome.

Pathogen	Location		Test	Cases		Controls		Odds Ratio (95% CI)	*p*-Value	Reference
	Country	Area (Year)		N	%	N	%			
*Onchocerca volvulus*	South Sudan	Lui (2001)	SSM	39	89.7	31	48.3	9.2 (2.7–3.26)	0.00003	[12,18]
		Amadi (2001)	SSM	30	96.7	34	50	29 (3.5–237.7)	-	[18]
		Lui (2002)	SSM	13	92.3	16	43.7	15.4 (1.6–148.8)	0.008	[12,18]
		Maridi and Witto (2011)	SSM	38	76.3	38	47.4	3.2 (1.2–8.7)	0.02	[30]
	Uganda	Kitgum (2009)	SSM	45	71.1	39	53.9	1.11 (0.37–3.27)	-	[31]
			Ov16 IgG	39	66.7	44	31.8	3.14 (1.08–9.13)	-	
			OvFAR/MSA	39	94.9	41	48.8	14.4 (2.65–78.3)		
		Kitgum and Pader (2016/17)	Ov16 IgG	154	93.5	153	54.9	8.79 (4.15–18.65)	0.001	[37]
*Mansonella perstans*	South Sudan	Lui (2001)	BM	39	41	31	9.6	3.2	0.005	[12]
		Amadi (2001)	BM	30	66.6	34	50			
*Loa loa*	South Sudan	Lui and Amadi (2001)	BM	69	0	65	0	-	-	[12]
*Wuchereria bancrofti*	South Sudan	Lui (2001)	BM	39	0	31	9	-	0.47	[12]
		Amadi (2001)		30	0	34	7.6	-		
*Trypanosoma brucei*	South Sudan	Lui (2021)	CATT	39	12.8	31	9.6	0.84	0.94	[12]
		Amadi (2001)		30	0	34	5.8			
	Uganda	Kitgum (2009)	CATT	36	0	40	0	-	-	[31]
*Taenia soleum*	Uganda	Kitgum (2009)	Antibody	36	0	40	0	-	-	[31]
Measles virus	Uganda	Kitgum (2009)	Past history		23.5		6.1	3.3 (0.8–13.6)		[31]
			PCR	16	0	0	-	-	-	
	South Sudan	Lui and Amadi (2002)	Past history	13	15.38	19	58	0.13	0.025	[12]
	Uganda	Kitgum (2014)	Past history	50	100	50	-	6 (1.02–113)	0.047	[35]
Hepatitis E virus		Kitgum (2009)	IgM	38	31.6	31	16.1	1.45 (0.37–5.58)	-	[31]
			IgG	38	26.3	30	33.3	0.81 (0.24–2.75)	-	

SSM: skin snip microscopy; BM: blood microscopy; CATT: card agglutination test.

**Table 4:** Case–control studies that studied nutritional deficiencies in nodding syndrome.

Micronutrient	Location		Cases		Controls		Odds Ratio (95% CI)	*p*-Value	Reference
	Country	Area (Year)	N	%	N	%			
Vitamin B6 (P5P)	Uganda	Gulu & Amuru district (2013)	66		73		7.22 (2.24–23.26)	0.001	[38]
	Uganda	Kitgum (2009)	49	73	42	64	1.22 (0.41–3.59)	-	[31]
	Uganda	-	3	100	5	100	-	-	[39]
Vitamin A	Uganda	Kitgum (2009)	25	40	12	33	2.15 (0.41–11.12)	-	[31]
Vitamin B12	Uganda	Kitgum (2009)	25	8	12	8	1.46 (0.09–22.82)	-	[31]
Folate	Uganda	Kitgum (2009)	11	9	9	0	-	-	[31]
Zinc	Uganda	Kitgum (2009)	17	47	12	67	0.72 (0.13–3.94)	-	[31]
Selenium	Uganda	Kitgum (2009)	17	100	12	100	-	-	[31]

P5P: pyridoxal-5-phosphate; PL: plasma level. Note: all nutrients were measured as plasma levels.

**Table 5:** Case–control studies reporting associations between toxins and nodding syndrome.

Toxins	Location		Test	Cases		Controls		Odds Ratio (95% CI)	*p*-Value	Reference
	Country	Area (Year)		N	%	N	%			
Mouldy maize	Uganda	Kitgum (2014)	DtH	50		50		4.33 (1.4–18.9)	0.009	[35]
Maize	Uganda	Kitgum (2014)	DtH	50		50		4 (1.0–26.5)	0.05	[35]
Emergency/relief food supplies	Uganda	Kitgum (2014)	DtH	47		50		4 (1.3–17.6)	0.016	[35]
		Gulu and Amuru (2016)	DtH	40	67	18	27	4.05 (1.23–13.28)	0.021	[38]
Red/brown sorghum	South Sudan	Mundri (2002)	DtH	13	54	19	16	6.22 (1.2–32.3)	0.049	[12]
	Uganda	Kitgum (2009)	DtH	-	98	-	100	1.3 (0.0–125.9)	-	[31]
Spoiled relief food	Uganda	Kitgum (2009)	DtH	-	43	-	47	0.3 (0.1–1.3)	-	[31]
Seeds meant for planting	Uganda	Kitgum (2009)	DtH	-	61	-	65	0.6 (0.1–2.3)	-	[31]
	South Sudan	Mundri (2002)	DtH	-	-	-	-	5 (0.82–30.4)	0.11	[12]
River fish	Uganda	Kitgum (2009)	DtH	-	96	-	100	0.3 (0.0–11.6)	-	[31]
Insects	Uganda	Kitgum (2009)	DtH	-	41	-	33	0.8 (0.2–2.9)	-	[31]
Rodent brain	Uganda	Kitgum (2009)	DtH	-	55	-	51	1.8 (0.3–12.3)	-	[31]
Baboon brain	South Sudan	Mundri (2002)	DtH	-	-	-	-	3 (0.63–14.2)	0.25	[12]
Baboon meat	South Sudan	Mundri (2002)	DtH	-	-	-	-	4.5 (0.97–20.8)	0.07	[12]
Crushed roots as traditional medicines	Uganda	Kitgum (2009)	DrH	-	39	-	16	5.4 (1.3–22.1)	-	[31]
	Uganda	Kitgum (2014)	DrH	50	-	50	-	1.29 (0.47–3.6)	0.617	[35]
Crushed leaves	Uganda	Kitgum (2009)	DrH	-	8	-	2	3.4 (0.2–45.8)	-	[31]
Crushed flowers	Uganda	Kitgum (2009)	DrH	-	0	-	2	0.9 (0.1–5.6)	-	[31]
Inhaled medicines	Uganda	Kitgum (2009)	DrH	-	2	-	0	0.2 (0.0–1.5)	-	[31]
Exposure to chemicals from munitions	Uganda	Kitgum (2009)	EH	-	70	-	51	13.9 (1.4–135)	-	[31]

DtH: dietary history; DrH: drug history; EH: exposure history.

**Table 6:** The 2013 modified consensus case definition for NS.

Suspected Case:	Reported Head Nodding in a Previously Healthy Person. Head Nodding Is Defined as Repetitive, Involuntary Drops of the Head towards the Chest on Two or More Occasions
Probable case	Suspected case of head nodding, with one major criterion plus at least one minor criterion *Major criteria:* 3–18 years of age at the onset of nodding *Minor criteria:* Other neurological abnormalities (cognitive decline, school dropout due to cognitive/behavioural problems, other seizures or neurological abnormalities) Clustering in space or time with similar cases Triggered by food or cold weather Stunting or wasting Psychiatric manifestations
Confirmed case	Probable case, with documented head-nodding episodes based on: Observation and recording by a trained healthcare worker, or A videotaped head-nodding episode, or Video/EEG/EMG documenting head nodding as atonic seizures

## Data Availability

All data are included in this manuscript. There is no other data involved in this study.
